# The Reverse Backlash: How the Success of Populist Radical Right Parties Relates to More Positive Immigration Attitudes

**DOI:** 10.1093/poq/nfad052

**Published:** 2023-12-12

**Authors:** James Dennison, Alexander Kustov

**Affiliations:** Researcher, School of Politics, Philosophy, Language, and Communication Studies, University of East Anglia, Norwich, UK; and Part-Time Professor, Migration Policy Centre, European University Institute, Florence, Italy; Assistant Professor, Department of Political Science and Public Administration, University of North Carolina at Charlotte, Charlotte, NC, US

## Abstract

What is the relationship between the electoral success of populist radical right parties (PRRPs) and public attitudes toward immigration? Previous research suggests that PRRP success can lead to more negative attitudes due to the breaking down of antiprejudice norms and more prominent anti-immigration party cues. However, we argue that greater PRRP success could have a positive relationship with immigration attitudes, reflecting negative partisanship, polarization, and a desire to reemphasize antiprejudice norms, which we call a “reverse backlash effect.” Using the best available electoral and public opinion data across the last thirty years in twenty-four European countries, our TSCS analyses show the predominance of such “reverse backlash effects” across several operationalizations of PRRP success. Our argument has important consequences for the understanding of possible PRRP effects on public opinion, as well as attitudinal formation via party cueing and social norms more generally.

## Introduction

In recent decades, populist radical right parties (PRRPs) have secured increasing electoral strength across Europe and beyond. While there has been significant research on how such parties cue widespread anti-immigration sentiments to improve their electoral fortunes, it is still unclear whether they actually change these very sentiments. The so-called “immigration backlash” is usually conceptualized as a populist response to proimmigration policy advancements or increasing immigration (Kustov 2023), but one can also imagine an analogous “reverse backlash” to populist electoral success in terms of improving public attitudes toward immigration, especially given the increasing polarization on the issue. The recent prominent examples of such a dynamic are the seeming proimmigration reactions of the US public to the election of Donald Trump (Collingwood et al. 2018) or the UK public in the aftermath of Brexit (Schwartz et al. 2021). In this paper, we test whether these notable cases of “reverse backlash” can be generalized to a larger set of the populist radical right successes across countries.

What is the relationship between the electoral success of PRRPs and public attitudes toward immigration? Intuitively, we might expect the increased success of populist radical right parties to have a negative effect on public attitudes to immigration via two related mechanisms: the increased ability of PRRPs to cue anti-immigration attitudes in the general population via greater resources, attention, and legitimacy (Zaller 1992; Hellwig and Kweon 2016; Harteveld, Kokkonen, and Dahlberg 2017; Vrânceanu and Lachat 2021) and the breaking of social stigmas related to open expression of anti-immigration sentiment (Rooduijn et al. 2016). Moreover, Abou-Chadi and Krause (2020; Van Spanje 2010) show that when radical right parties enter parliament, it moves mainstream parties toward more anti-immigration positions (meaning that at the aggregate level the growth of PRRPs may also lead to anti-immigration sentiment *via* other parties’ cueing efforts). At the same time, Valentim (2021) shows that PRRP entry to parliament erodes social norms so that *their voters* see their views as legitimized and are more willing to publicly express their intention to vote for such parties.

However, empirically, despite the rise of PRRPs over the last decades, attitudes to immigration have been shown to be stable over the long term (Kustov, Laaker, and Reller 2021). Moreover, as the electoral breakthroughs and successes of PRRPs became more widespread and acute across Europe in the aftermath of the 2015–2016 so-called “migration crisis,” there was not and has still not been a correspondent negative turn in attitudes to immigration. Indeed, according to some sources of data and as measured by certain questions, attitudes to immigration have become more positive in several European countries (Dennison and Geddes 2019; Banai, Votta, and Seitz 2022).

In this research note, we argue and empirically demonstrate that PRRP electoral success is associated with greater positivity in overall public attitudes via what we dub a “reverse backlash effect.” If present, this relationship might reflect the desire among the majority of citizens to reemphasize antixenophobic social norms and stigma toward PRRPs’ key policies (e.g., Mendes and Dennison 2021), as well as express negative partisanship toward such parties (e.g., Meléndez and Kaltwasser 2021). It may also reflect a reduction in ambivalence toward the topic of immigration via higher issue salience and polarization more generally, with a majority of those who were ambivalent moving toward proimmigration positions (Lindstam, Mader, and Schoen 2021). Finally, this “reverse backlash effect” may also be conceptualized as a form of thermostatic public opinion. Rather than the thermostat measuring feeling toward particular policies (Wlezien 1995), it would be toward the direction and state of the party system and societal norms regarding what constitutes an acceptable political opinion.

Findings related to similar albeit less generalizable events lend plausibility to our central proposition. Scholars (Schwartz et al. 2021; Solodoch 2021) have shown that the UK’s vote to leave the EU (“Brexit”) led to an increase in self-reported *positivity* toward immigrants, theorized as resulting from satisfaction that immigration “control” had been “restored” and a desire to distance oneself from accusations of xenophobia and racism following the controversial referendum campaign. The latter point is grounded in the Motivation to Control Prejudice theory, which posits that “individuals are averse to breaking antiprejudice norms and will deliberately seek to control actions, expressions or thoughts that can be deemed to violate these norms” (Fazio et al. 1986; Devine 1989; Devine et al. 1991; Czopp and Monteith 2003; Dovidio and Gaertner 2004; Blinder, Ford, and Ivarsflaten 2013; Schwartz et al. 2021, p. 1169). Crucially, the Motivation to Control Prejudice theoretically requires continuous self-reflection about one’s own views to external events to control and check manifestations of prejudice to avoid social sanction (Monteith et al. 2002; Blinder, Ford, and Ivarsflaten 2013). As such, the widely discussed and controversial rise in radical right support in Europe in the 2010s may plausibly have—like “Brexit”—triggered a psychological imperative for many individuals to redouble their support for immigration. Indeed, Blinder, Ford, and Ivarsflaten (2013, p. 845) argue that politicians or parties with “clear racist or fascist reputations” can be expected to “activate the antiprejudice norm” while the controversial leaders of PRRPs make individuals “*aware* that a norm is at stake, before they take the cognitive effort to control prejudice and adjust their response in accordance with it” (Harteveld, Kokkonen, and Dahlberg 2017, p. 372).

Finally, we may also expect the increased success of PRRPs to have no effect on public attitudes to immigration. Such attitudes have been shown to be stable at the aggregate level (Kustov, Laaker, and Reller 2021), theoretically the result of attitudes being formed in early life and reflecting deep-seated psychological predispositions and national and education socializing experiences (Kauff et al. 2013; Dinesen, Klemmensen, and Nørgaard 2016; García-Faroldi 2017; Dennison, Davidov, and Seddig 2020; McLaren and Paterson 2020; McLaren, Neundorf, and Paterson 2021). Therefore, attitudinal changes that do occur happen gradually at the aggregate level due to generational replacement, as those socialized in more precipitous conditions for immigration sympathy (e.g., heterogenous societies, “universalist” tertiary education) enter adulthood.

We test these three possible alternatives regarding the possible relationship of PRRP success with anti-immigration attitudes (positive, negative, or null) using the standard fixed-effects TSCS specifications and the best available electoral and public opinion data across the last several decades in Europe. To capture PRRP success, we look at the vote and the seat shares won by the populist right as defined by the Popu-List or the Timbro Index data. To capture immigration attitudes, we aggregate the relevant public opinion items from major sources.

## Data and Methods

Since the electoral success of PRRPs reflects the preferences and priorities of voters alongside other unobserved factors, any cross-sectional associations (or the lack thereof) are likely subject to endogeneity concerns, including reverse causality and omitted variable bias. The standard econometric solution to these problems is to produce plausible causal estimates by utilizing panel data with unit fixed-effects regression models, which can account for unobserved country-specific time-invariant confounding factors—such as historic, “constant” PRRP success and other fixed structural, historic, and long-term factors—under a few reasonable assumptions.^1^ The main identifying assumption of such an approach here is that immigration attitudes would have developed similarly in countries with and without the observed PRRP success had it not happened. As such, our approach cannot solve all potential sources of endogeneity, such as omitted variables that account for both PRRP success and immigration attitudes or reverse causality—though theoretically we believe it is unlikely that PRRPs can capitalize on proimmigration attitudes.

To test for the potential reverse backlash, we have thus gathered a TSCS dataset linking the best available electoral and public opinion data at the country-year level across twenty-four European countries (1989–2017).^2^ As for our main dependent variable, we construct an aggregate country-year measure of public anti-immigration attitudes comprised from the two most comprehensive data sources on the topic. We primarily rely on the “immigration conservatism” index compiled by Caughey, O’Grady, and Warshaw (2019), which aggregates major cross-national public opinion survey questions on immigration using an item response theory model. We then standardize and average it with the similar “immigration mood” index created by Claassen and McLaren (2021), which uses a slightly different aggregation methodology and a range of country-year cases (r = 0.81). Our results are robust to the use of these indices separately (see Supplementary Material tables S2 and S3).

As for our independent variables, we primarily rely on the Popu-List project combined with ParlGov data, which provides a comprehensive measure of the share of votes and seats won by the “far-right populist” parties in European national legislatures in each country-year since the last elections. These are exactly the parties that have been either explicitly anti-immigration or otherwise attractive to the anti-immigration electorate (Rooduijn et al. 2019). As a robustness check, we also look at the conceptually similar vote share of “right-wing populist” parties as coded by the Timbro Authoritarian Populism Index (Heinö 2016; r = 0.92). Some specifications also include common (lagged) control variables, including the share of immigrant population, unemployment rate (log), and GDP per capita (log). All major dependent and independent variables have been standardized to vary from 0 to 1. All coefficient estimates are reported using heteroskedasticity-robust standard errors.^3^ For more details on variables and their construction, see the Supplementary Material.

## Results and Analysis

First, we consider the general relationship between the PRRP success and anti-immigration attitudes across countries and time. In line with the conventional wisdom, the naïve cross-sectional correlation between these variables in the pooled data is consistently positive and varies from 0.06 to 0.25 depending on the operationalization (see Supplementary Material figure S1).

**Figure 1.**
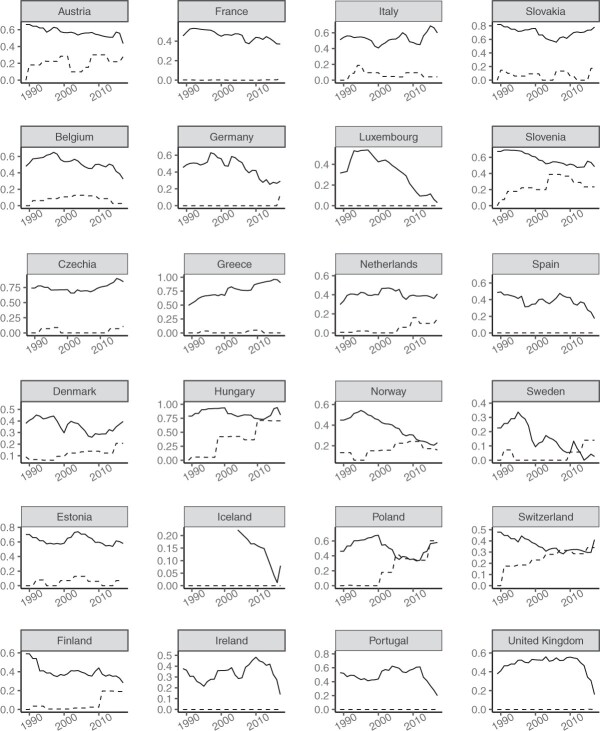
The trajectories of PRRP success and anti-immigration attitudes across Europe (1989–2017). Higher values on the y-axis correspond to higher PRRP success or anti-immigration attitudes. Dashed lines indicate various measures of PRRP success. Solid lines indicate various measures of anti-immigration attitudes. For variable descriptions, see the Supplementary Material.

We then plot the historical trajectories of PRRP success and anti-immigration attitudes within each European country (see figure 1). As can be seen, despite their positive cross-sectional correlation and their conflation in the popular discourse, these dependent and independent variables rarely go together across time regardless of their operationalization. If anything, there seems to be a negative correlation between these two phenomena within most of the countries.

We thus further take advantage of the time-series cross-sectional data to fit a set of fixed-effects linear regressions to answer the following question: “As PRR success changes within a certain country, how does it relate to the changes in aggregate immigration attitudes over time?”

Table 1 summarizes our main empirical results. As can be seen, the success of PRRPs has a consistent negative relationship with anti-immigration attitudes. This is true regardless of PRRP success operationalization or a particular model specification. In terms of substantive size of this relationship, a one standard deviation increase in the seat and vote share of PRRPs is associated with a 0.16 and 0.2 standard deviation increase of proimmigration sentiments within countries, respectively. Overall, this evidence is consistent with the hypothesized reverse backlash effects of PRRP success, at least in the aggregate.

**Table 1.** Populist right electoral success and anti-immigration attitudes. The table shows the relationship between PRRP success and anti-immigration attitudes. For variable descriptions, see the Supplementary Material. Heteroskedasticity-robust standard errors are given in parentheses.

	Anti-immigration attitudes								
	(1)	(2)	(3)	(4)	(5)	(6)	(7)	(8)	(9)
Far-right populist (seat share)	−0.226	−0.158	−0.135						
	(0.043)	(0.043)	(0.046)						
Far-right populist (vote share)				−0.301	−0.233	−0.208			
				(0.048)	(0.047)	(0.050)			
Right-wing populist (vote share)							−0.266	−0.167	−0.136
							(0.042)	(0.041)	(0.042)

Country FE	Yes	Yes	Yes	Yes	Yes	Yes	Yes	Yes	Yes
Controls	No	Yes	Yes	No	Yes	Yes	No	Yes	Yes
Year FE	No	No	Yes	No	No	Yes	No	No	Yes
Observations	681	660	660	681	660	660	666	660	660
Adjusted R^2^	0.794	0.824	0.821	0.798	0.826	0.823	0.793	0.824	0.821

To further check the robustness of these results, we conduct several additional empirical tests with little change in the substantive finding. First, we replicate the specifications from table 1/Supplementary Material table S2 using the “economic conservatism index” from Caughey, O’Grady, and Warshaw (2019) as a placebo test, which expectedly shows no relationship with the PRRP success (see Supplementary Material table S4). Second, given that populist voting is not observed every year, we replicate our specifications at the more empirically appropriate, but less statistically powered, country-election level (see Supplementary Material table S5).

## Discussion

How does the success of populist parties relate to voters’ immigration views? Given that right-wing populists use anti-immigration rhetoric in their political campaigns, it is tempting to think that, if anything, they can only make the electorate more anti-immigration when they emerge, gain support, and come to power. After all, in theory the rise of PRRPs should give them greater capacity to cue supporters and the general public (Zaller 1992; Hellwig and Kweon 2016; Harteveld, Kokkonen, and Dahlberg 2017; Vrânceanu and Lachat 2021) as well as an already observed (Rooduijn, Van der Brug, and De Lange 2016; Valentim 2021) reduction in stigma toward self-reporting support for such parties, which could theoretically also affect self-reporting antipathy or opposition toward immigration.

However, populism is polarizing (Bischof and Wagner 2019; Dai and Kustov 2022). While radical right views and parties carry significant social stigma and evoke negative partisanship (Meléndez and Kaltwasser 2021; Mendes and Dennison 2021), it is also possible that there may be a reverse backlash among voters who want to distance themselves personally from such parties and views. Indeed, voters may wish to halt the progress of such parties, their views, and their increased acceptability, or—particularly among moderates—see the rise of such parties as a signal that enough has been done to voice concerns about (perhaps selected aspects of) immigration policies or realities. Furthermore, the rise of such parties may lead to greater cognition about the issue of immigration and thus a reduction in attitudinal ambivalence (Lindstam, Mader, and Schoen 2021), with a majority of those who were ambivalent leaning toward proimmigration positions, particularly relative to the now higher-profile PRRPs.

In this paper, we tried to use the best available data to examine which possibility is more likely. While it is possible that both effects can be happening at the same time, the evidence is more consistent with the “reverse backlash effect.” That is, when PRRPs become increasingly successful, a significant proportion of the electorate “thermostatically” reacts to these developments by adjusting their stated preferences toward immigration in a positive direction. Given the general stability of immigration attitudes, however, it is important not to overstate the magnitude of these possible effects. Still, the available evidence strongly suggests that the populist success does not make the electorate more anti-immigration in the aggregate.

Of course, our study is not without limitations. In terms of endogeneity, one can still reasonably worry about reverse causality and omitted variable bias. As such, our findings should be regarded as associational rather than causal. Although we cannot rule out other confounding factors, theoretically there are few reasons to believe that the relationship reflects more positive attitudes to immigration causing greater PRRP success. In fact, the extent to which PRRPs rely on negative immigration attitudes when they come to power would only bias the regression estimates *against* finding any contemporaneous “reverse backlash effects,” contrary to what we find. Furthermore, the PRRP success may have an impact only on some voters, and some of these impacts may even cancel each other out in the aggregate (e.g., when PRRP success increases or decreases anti-immigration attitudes among PRRP and non-PRRP voters). Notably, if the greater positivity toward immigration were equal among PRRP and non-PRRP voters, it would undermine the stigma mechanism, and instead support the thermostatic interpretation. Future research may thus benefit from using individual-level longitudinal data to explore the potential heterogeneous effects on different types of voters and the related mechanisms in more detail, as well as exploring the more credible identification strategies to isolate the causal effects of PRRP success. Finally, our current dataset ends in 2017, after which the rise of PRRPs has, in many countries, stopped or even gone into reverse while public attitudes to immigration have tended to gradually become more positive (Banai, Votta, and Seitz 2022). This suggests that, if tested again in the future with the use of more recent data, the stipulated relationship may be stronger or, at the very least, is unlikely to disappear.

These limitations notwithstanding, it is important to know that the relationship between PRRP success and anti-immigration attitudes is in fact negative across the broadest sample of electoral contexts. This descriptive finding has several implications. First, social stigmas may mediate the effects of PRRP cueing on attitudes, not only precluding immigration backlash but also potentially reversing it. Second, just because the key determinants of attitudes to immigration—notably early life socialization and psychological predispositions—are “deep-seated” and have a constant component, that does not mean that they are not dynamic and subject to mediating exogenous factors. Third, the policy preferences of political supply (i.e., parties) have repeatedly been shown to be primarily affected by the policy preferences of political demand (i.e., voters), rather than vice versa. We offer a case whereby political demand seems to be related to political supply, albeit not in the manner that the political supply might prefer. Finally, although the electoral breakthroughs of PRRPs were for many countries a radical change from postwar or posttransition party systems, which was distressing or even traumatic for many individuals, it does not seem that social norms and stigmas around xenophobic attitudes and radical right parties were entirely “broken” by these events. Instead, it seems that they survived and found new forms of expression in most citizens who reject such norms.

## Supplementary Material

nfad052_Supplementary_Data

## Data Availability

Replication data and documentation are available at https://dataverse.harvard.edu/dataset.xhtml?persistentId=doi:10.7910/DVN/EPIQHH.

## Footnotes

1 The common two-way fixed-effects models have been recently criticized in the literature for their unclear counterfactual interpretation (Kropko and Kubinec 2020; Imai and Kim 2021). We thus use the simpler unit FEs as our starting specification but also report two-way FEs as a robustness check.

2 Information on data sources and corresponding response rates is included in the SI.

3 Given that the PRRP success is “assigned” at the level of countries, we also apply the more conservative country-clustered standard errors across all specification with no change in the substantive results (see table S1).
