# Supplementary material for: The Reverse Backlash: How the Success of Populist Radical Right Parties Relates to More Positive Immigration Attitudes
Source: Public Opin Q. 2023 Dec 12;87(4):1013–24. doi: 10.1093/poq/nfad052 (PMC11550558; doi:10.1093/poq/nfad052)
Supplement: nfad052_Supplementary_Data [file nfad052_supplementary_data.pdf]

Supplementary Material:

The Reverse Backlash: How the Success of Populist Radical Right Parties Relates to More Positive Immigration Attitudes

James Dennison
University of East Anglia
School of Politics, Philosophy, Language, and Communication Studies
Norwich, NR4 7TJ, UK

European University Institute
Migration Policy Centre
Via della Badia dei Roccettini, 9, Fiesole, 50014, Italy
james.dennison@eui.eu

Alexander Kustov
University of North Carolina at Charlotte
Department of Political Science and Public Administration
9201 University City Blvd., Charlotte, NC 28223, USA
akustov@uncc.edu

Table of Content:

Variable Descriptions

Methodology

Figures and Tables

Variable Descriptions

PRRP success

- *Far-right populist (seat share, 0-1)* indicates the share of seats won by "far-right populist" parties in each country-year since the last elections in a national legislature as coded by Popu-List (Rooduijn et al2019).
- *Far-right populist (vote share, 0-1)* indicates the share of votes cast for "far-right populist" parties in each country-year since the last elections in a national legislature as coded by Popu-List (Rooduijn et al2019).
- *Right-wing populist (vote share, 0-1)* measures the share of votes cast for "right-wing populist" parties in each country-year since the last elections in a national legislature as coded by the Timbro Authoritarian Populism Index (Heino, 2016).

Immigration attitudes

- *Anti-immigration attitudes (0-1)* indicate the immigration-related restrictiveness of the electorate in a given country/year based on a combination (average) of "immigration conservatism" index compiled by Caughey et al. (2019) and the "immigration mood" index compiled by Claassen and McLaren (2021). Both indices use slightly different methodologies to aggregate major public opinion questions on immigration. The Caughey et al. index uses an ordinal group-level item response theory model and the Claassen and McLaren index uses a slightly different dynamic latent variable model to summarize most of the available survey questions about immigration from the European Social Survey (ESS), International Social Survey Program (ISSP), World Values Survey (WVS), the Pew Global Attitudes Survey, and Eurobarometer.
- For the election-level analysis, the measure is aggregated over the entire between-election periods (2-5 years depending on the country/period).

Other variables

- *Economic attitudes (0-1)* indicate the (absolute) voter preferences on economic policies as based on the "economic conservatism" index complied by Caughey et al. (2019).
- Control variables include *immigration stocks*, *unemployment rate (log)*, and *GDP per capita (PPP, log)* derived from the publicly available OECD and the World Bank data.

Methodology

Our measure of anti-immigration attitudes indicates the (estimated) immigration-related restrictiveness of the electorate in a given country/year based on a combination (average) of "immigration conservatism" index compiled by Caughey et al. (2019) and the "immigration mood" index compiled by Claassen and McLaren (2021). In doing so, we rely on the (subset of) aggregate country-year-level estimates of immigration attitudes based on statistical models derived from the previously compiled hundreds of publicly available sources. Both indices use slightly different methodologies to aggregate major public opinion questions on immigration. The Caughey et al. index uses an ordinal group-level item response theory model and the Claassen and McLaren index uses a slightly different dynamic latent variable model to summarize most of the available survey questions about immigration from the European Social Survey (ESS), International Social Survey Program (ISSP), World Values Survey (WVS), the Pew Global Attitudes Survey, and Eurobarometer.

The public opinion estimates we use are based on scores of separate publicly available surveys from different countries, each with its own response rates. As such, for the full list of sources, please refer to the appendices of Caughey et al. (2019) and Claassen and McLaren (2021) available here, respectively:
https://static.cambridge.org/content/id/urn:cambridge.org:id:article:S0003055419000157/resource/name/S0003055419000157sup001.pdf
https://static.cambridge.org/content/id/urn:cambridge.org:id:article:S0007123421000260/resource/name/S0007123421000260sup001.pdf
While the authors of these original estimates do not explicitly list the response rates of all the hundreds of surveys they used to create those estimates, interested readers can find the list of surveys they used in the linked appendices for the papers introducing these data. Interested readers can also go to the official website of those publicly available survey providers such as the European Social Survey and find information about the response rate for a particular survey of interest.

Figures and Tables

Figure S1: Populist Right Electoral Success and Anti-Immigration Attitudes

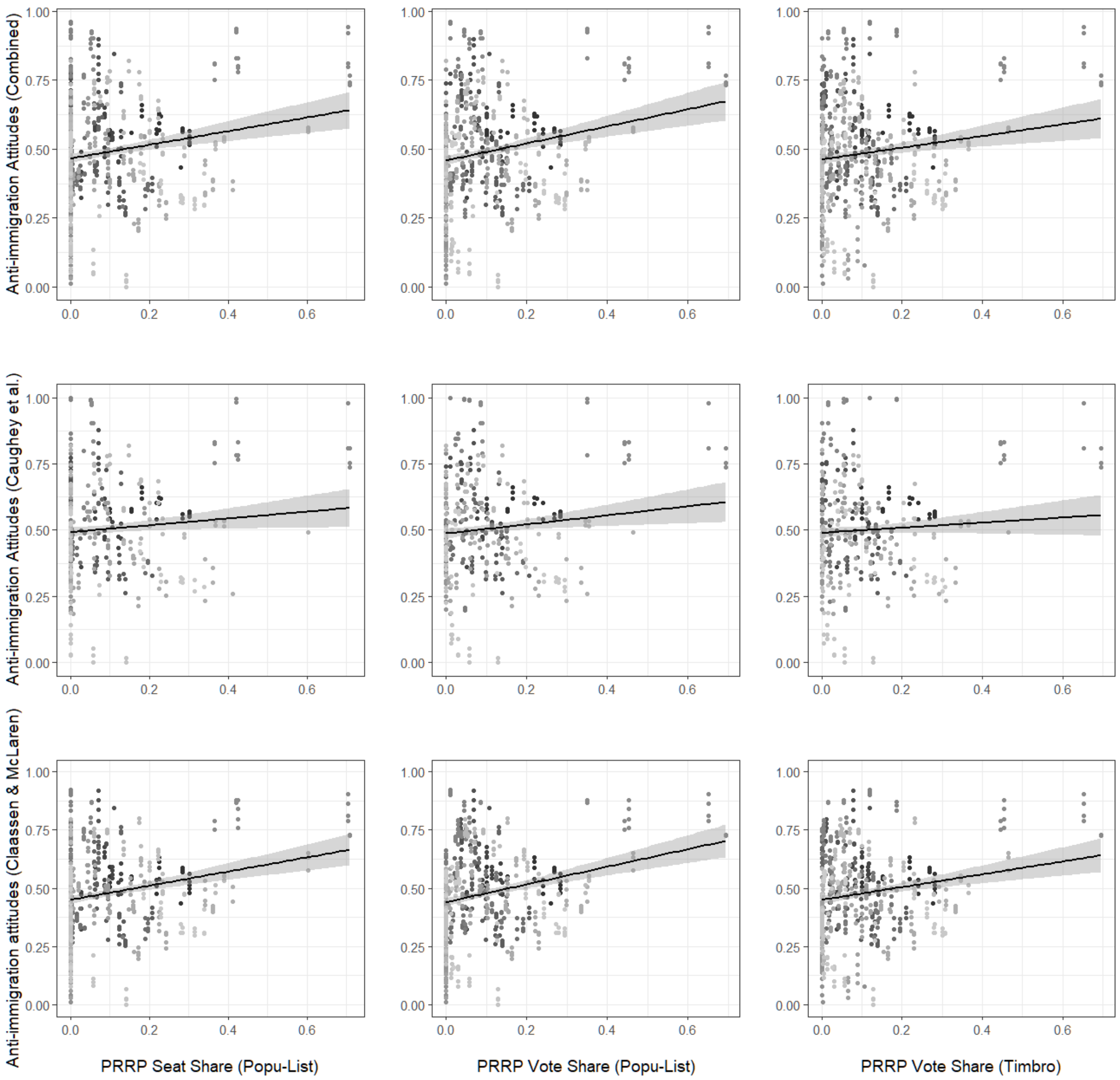

Notes: The figure depicts bivariate relationships between the various measures of PRRP success and anti-immigration attitudes at the country-year level. All points are shaded by country. For variable descriptions, see Appendix.

Table S1: Populist Right Electoral Success and Anti-Immigration Attitudes (Robustness Check I)

	Anti-immigration attitudes								
	(1)	(2)	(3)	(4)	(5)	(6)	(7)	(8)	(9)
Far-right populist (seat share)	-0.226**	-0.158*	-0.135						
	(0.074)	(0.066)	(0.069)						
Far-right populist (vote share)				-0.301**	-0.233*	-0.208*			
				(0.096)	(0.091)	(0.092)			
Right-wing populist (vote share)							-0.266**	-0.167*	-0.136*
							(0.102)	(0.078)	(0.067)
Country FE	Yes	Yes	Yes	Yes	Yes	Yes	Yes	Yes	Yes
Controls	No	Yes	Yes	No	Yes	Yes	No	Yes	Yes
Year FE	No	No	Yes	No	No	Yes	No	No	Yes
Observations	681	660	660	681	660	660	666	660	660
Adjusted R^2	0.794	0.824	0.821	0.798	0.826	0.823	0.793	0.824	0.821

Notes: The table shows the relationship between PRRP success and anti-immigration attitudes. For variable descriptions, see Appendix. Robust standard errors clustered by country are given in parentheses: ∗p<0.05; ∗∗p<0.01; ∗∗∗p<0.001.

Table S2: Populist Right Electoral Success and Anti-Immigration Attitudes (Robustness Check II)

	Anti-immigration attitudes (Caughey et al.)								
	(1)	(2)	(3)	(4)	(5)	(6)	(7)	(8)	(9)
Far-right populist (seat share)	-0.283***	-0.231***	-0.235***						
	(0.054)	(0.057)	(0.062)						
Far-right populist (vote share)				-0.363***	-0.323***	-0.325***			
				(0.060)	(0.063)	(0.068)			
Right-wing populist (vote share)							-0.271***	-0.179**	-0.186**
							(0.054)	(0.055)	(0.057)
Country FE	Yes	Yes	Yes	Yes	Yes	Yes	Yes	Yes	Yes
Controls	No	Yes	Yes	No	Yes	Yes	No	Yes	Yes
Year FE	No	No	Yes	No	No	Yes	No	No	Yes
Observations	616	595	595	616	595	595	601	595	595
Adjusted R^2	0.766	0.783	0.776	0.771	0.786	0.779	0.762	0.779	0.773

Notes: The table shows the relationship between PRRP success and anti-immigration attitudes (as measured by Caughey et al. index). For variable descriptions, see Appendix. Heteroskedasticity-robust standard errors are given in parentheses: ∗p<0.05; ∗∗p<0.01; ∗∗∗p<0.001.

Table S3: Populist Right Electoral Success and Anti-Immigration Attitudes (Robustness Check III)

	Anti-immigration attitudes (Claassen and McLaren)								
	(1)	(2)	(3)	(4)	(5)	(6)	(7)	(8)	(9)
Far-right populist (seat share)	-0.201***	-0.139**	-0.042						
	(0.048)	(0.049)	(0.049)						
Far-right populist (vote share)				-0.289***	-0.216***	-0.108			
				(0.055)	(0.056)	(0.056)			
Right-wing populist (vote share)							-0.249***	-0.199***	-0.097*
							(0.042)	(0.044)	(0.044)
Country FE	Yes	Yes	Yes	Yes	Yes	Yes	Yes	Yes	Yes
Controls	No	Yes	Yes	No	Yes	Yes	No	Yes	Yes
Year FE	No	No	Yes	No	No	Yes	No	No	Yes
Observations	618	615	615	618	615	615	618	615	615
Adjusted R^2	0.772	0.803	0.820	0.775	0.805	0.821	0.776	0.806	0.821

Notes: The table shows the relationship between PRRP success and anti-immigration attitudes (as measured by Claassen and McLaren index). For variable descriptions, see Appendix. Heteroskedasticity-robust standard errors are given in parentheses: ∗p<0.05; ∗∗p<0.01; ∗∗∗p<0.001.

Table S4: Populist Right Electoral Success and Economic Attitudes (Placebo Test)

	Economic Conservatism (Caughey et al.)								
	(1)	(2)	(3)	(4)	(5)	(6)	(7)	(8)	(9)
Far-right populist (seat share)	-0.005	-0.047	-0.006						
	(0.043)	(0.048)	(0.046)						
Far-right populist (vote share)				-0.025	-0.081	-0.041			
				(0.048)	(0.056)	(0.056)			
Right-wing populist (vote share)							-0.043	-0.139*	-0.103
							(0.049)	(0.054)	(0.052)
Country FE	Yes	Yes	Yes	Yes	Yes	Yes	Yes	Yes	Yes
Controls	No	Yes	Yes	No	Yes	Yes	No	Yes	Yes
Year FE	No	No	Yes	No	No	Yes	No	No	Yes
Observations	532	511	511	532	511	511	517	511	511
Adjusted R^2	0.667	0.676	0.696	0.667	0.677	0.696	0.667	0.680	0.698

Notes: The table shows the relationship between PRRP success and economic attitudes (as measured by Caughey et al. index). For variable descriptions, see Appendix. Heteroskedasticity-robust standard errors are given in parentheses: ∗p<0.05; ∗∗p<0.01; ∗∗∗p<0.001.

Table S5: Populist Right Electoral Success and Anti-Immigration Attitudes (Election-level Analysis)

	Anti-immigration attitudes								
	(1)	(2)	(3)	(4)	(5)	(6)	(7)	(8)	(9)
Far-right populist (seat share)	-0.224***	-0.199**	-0.207^{+}						
	(0.065)	(0.069)	(0.125)						
Far-right populist (vote share)				-0.272***	-0.255**	-0.238			
				(0.082)	(0.096)	(0.152)			
Right-wing populist (vote share)							-0.198**	-0.146*	-0.114
							(0.077)	(0.074)	(0.102)
Country FE	Yes	Yes	Yes	Yes	Yes	Yes	Yes	Yes	Yes
Controls	No	Yes	Yes	No	Yes	Yes	No	Yes	Yes
Year FE	No	No	Yes	No	No	Yes	No	No	Yes
Observations	180	173	173	180	173	173	177	173	173
Adjusted R^2	0.801	0.838	0.839	0.802	0.839	0.838	0.794	0.835	0.834

Notes: The table shows the relationship between PRRP success and anti-immigration attitudes at the between-election level. For variable descriptions, see Appendix. Heteroskedasticity-robust standard errors are given in parentheses: $*p<0.05$; $**p<0.01$; $***p<0.001$.
